# Dissection vertébrale post-traumatique: résultats IRM

**DOI:** 10.11604/pamj.2017.26.78.11256

**Published:** 2017-02-20

**Authors:** Youssef Alaoui Lamrani, Mustapha Maaroufi

**Affiliations:** 1 Faculté de Médecine et de Pharmacie, Université Sidi Mohamed Ben Abdellah, Service de Radiologie, CHU Hassan II, Fès, Maroc

**Keywords:** Vertebral artery, arterial dissection, MRI, Vertebral artery, arterial dissection, MRI

## Image en médecine

IRM cervicale en coupe sagittale T2 montrant une contusion œdémateuse médullaire (étoile), associée à un hématome prévertébral (H). Les autres coupes T2 (b, c), et T2* (d) révèlent une thrombose de l’artère vertébrale (AV) droite (flèche). L’occlusion atteint le segment V3 et sans lésion ischémique intracrânienne. Monsieur D.L. âgée de 43 ans, a été victime d’un traumatisme cervical lors d’un accident de la voie publique. L’examen révèle une tétraparésie non expliquée par les données TDM qui montrent des fractures vertébrales irradiant vers les trous transverses C5 et C6 droits. Le recours en deuxième intention à l'IRM cervicale a révélé une contusion médullaire œdémateuse, avec occlusion de l’AV droite sur tout son trajet extra-crânien, et qui est le siège d’un hématome intramural suggérant une dissection vertébrale sous-jacente. Son caractère asymptomatique est liée à une AV gauche perméable avec un polygone de Willis fonctionnel assurant la suppléance du système vertébro-basilaire. Le caractère complet de l’occlusion à éviter toute complication thrombo-embolique. La dissection des AV extra-crâniennes est associée à des traumatismes de haute énergie et corrélée à la gravité du bilan lésionnel ostéo-médullaire. La clinique est masquée par d’autres lésions traumatiques à la phase aigue, et les symptômes sont d’apparition retardée. Ces derniers sont d’ordre ischémique de mécanisme hémodynamique ou thrombo-embolique. L’extension de la dissection en intracrânien peut causer une hémorragie méningée. Le traitement repose sur des anticoagulants ou antiagrégants en absence de lésions viscérales ou intracrâniennes sévères, le recours au traitement endovasculaire s’avère nécessaire en présence d’une menace ischémique dynamique.

**Figure 1:**
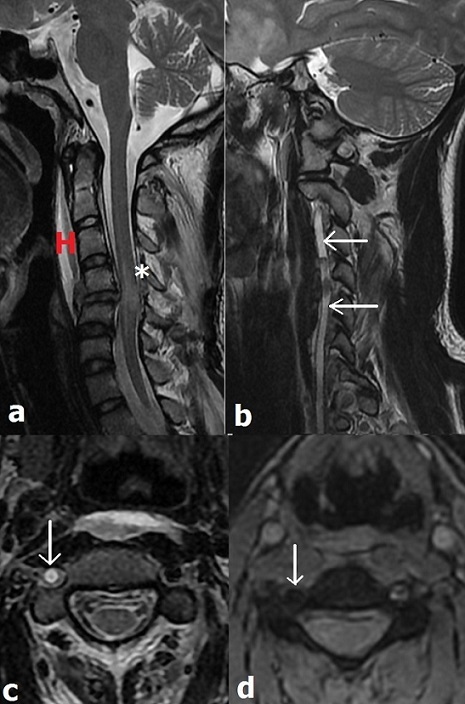
IRM cervicale en coupe sagittale T2 (a) montrant une contusion œdémateuse médullaire à la hauteur de C4-C5, associée à un hématome prévertébral (tête de flèche rouge) avec rupture du ligament longitudinal antérieur (tête de flèche blanche). Les autres coupes T2 (b, c), et T2+ révèlent une thrombose de l’artère vertébrale (AV) droite (flèches). L’occlusion atteint le segment V3. Les séquences intracrâniennes n’ont pas montré de lésion ischémique récente

